# Supplementary material for: Plasma Proteomic Analysis in Non-Small Cell Lung Cancer Patients Treated with PD-1/PD-L1 Blockade
Source: Cancers (Basel). 2021 Jun 22;13(13):3116. doi: 10.3390/cancers13133116 (PMC8268315; doi:10.3390/cancers13133116)
Supplement: Supplementary file 1 [file cancers-13-03116-s001.zip › cancers-1240811-supplementary.pdf]

Supplementary Materials

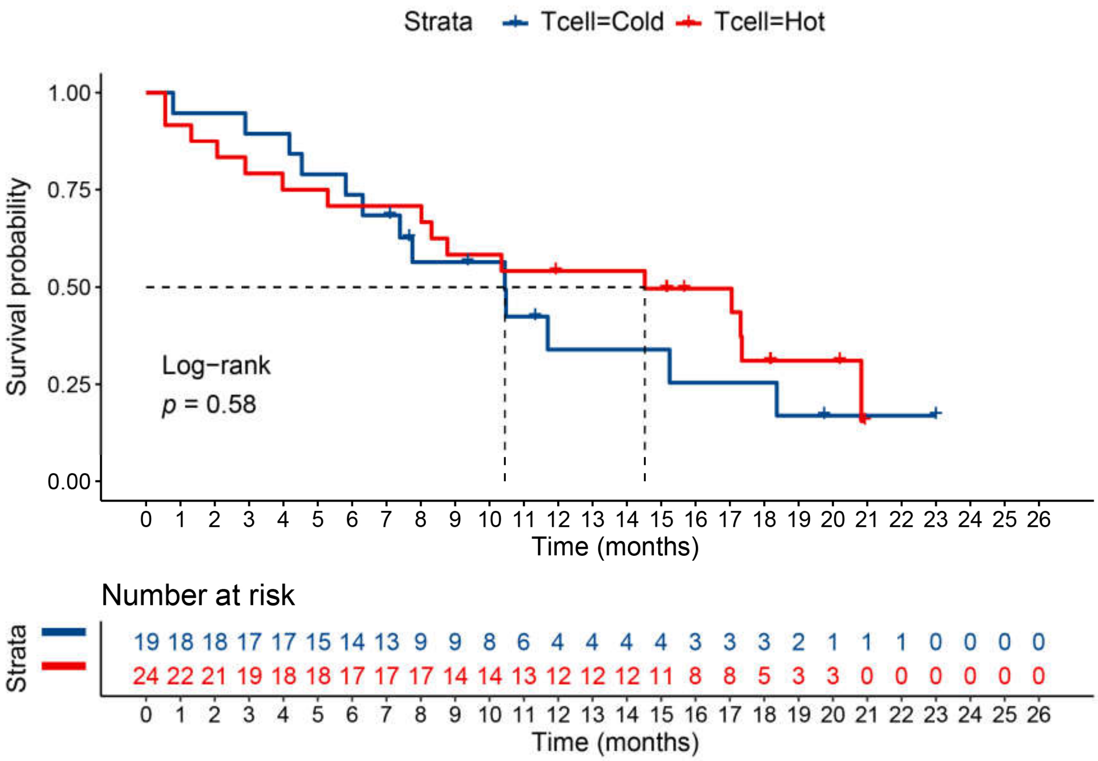

Figure S1. Kaplan–Meier overall survival curves based on hot and cold T cell activation signature. A log rank test was performed to compare the two groups.

Supervised clustering based on APC-related markers

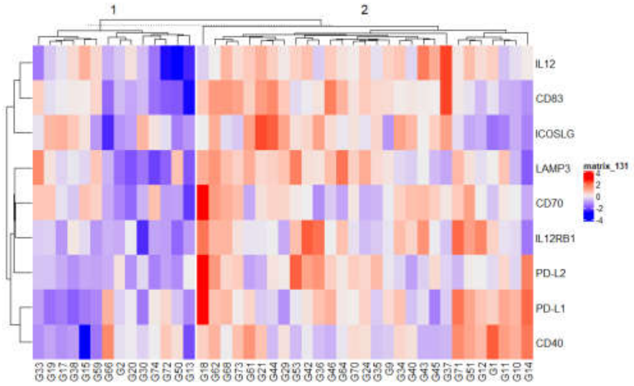

Supervised clustering based on Myeloid/angiogenesis-related markers

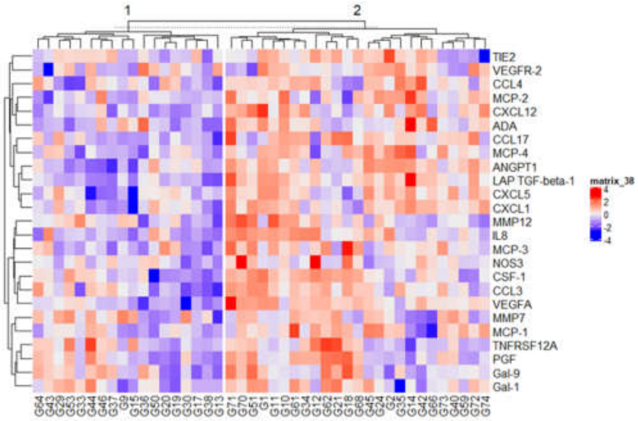

Supervised clustering based on tumor-related markers

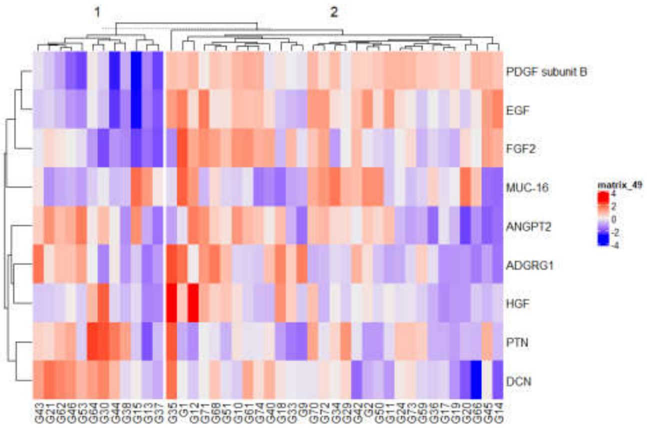

Figure S2. Hierarchal clustering analysis of protein expression. K-means clustering analysis of selected APC-related markers (9 proteins), myeloid/angiogenesis (25 proteins) and tumor-related markers (9 proteins) in the plasma.

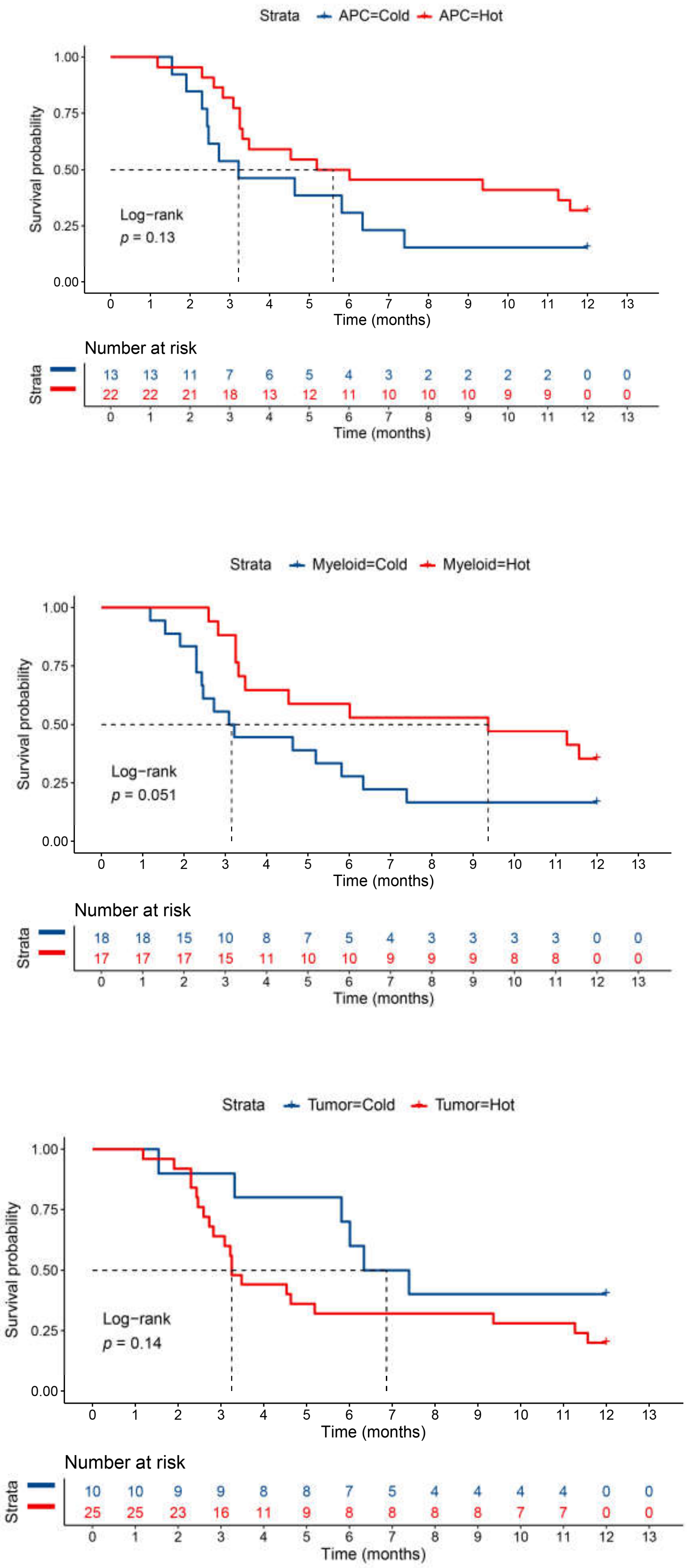

Figure S3. Progression-free survival (PFS) analysis of APC-related markers (9 proteins), myeloid/angiogenesis (25 proteins) and tumor-related markers (9 proteins) in the plasma signature (hot vs. cold). The log rank test was used to test for differences in survival.

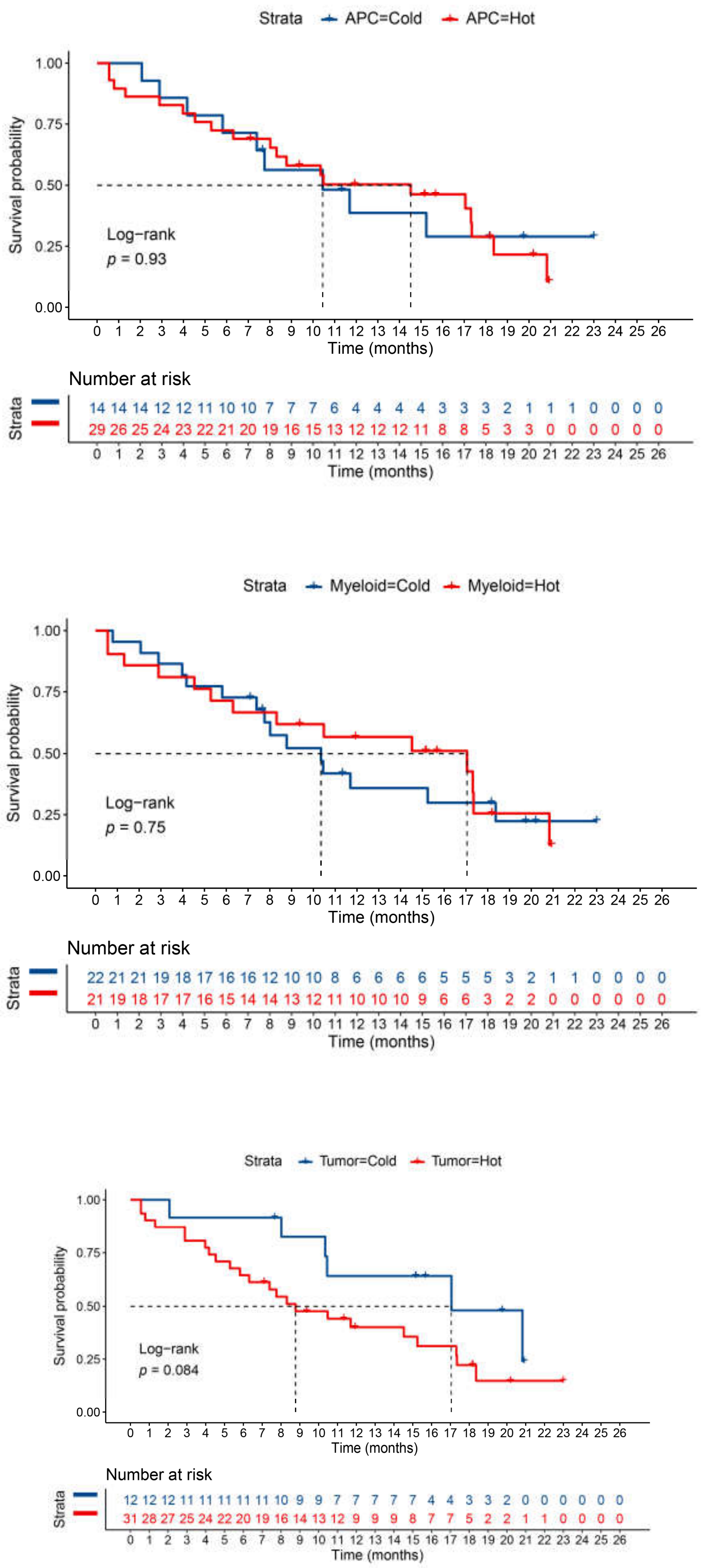

Figure S4. Overall survival (OS) analysis of APC-related markers (9 proteins), myeloid/angiogenesis (25 proteins) and tumor-related markers (9 proteins) in the plasma signature (hot vs. cold). The log rank test was used to test for differences in survival.

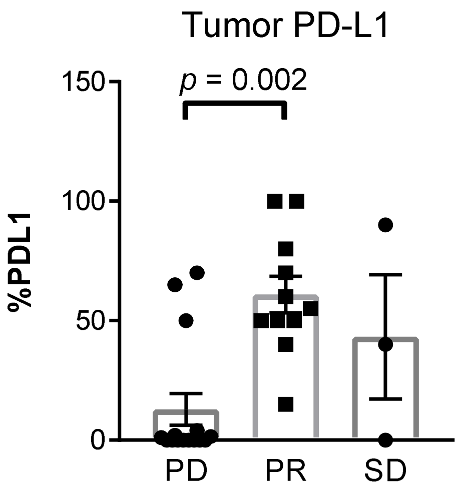

Figure S5. Comparison between patients with progressive disease (PD), spatial response (PD) and stable disease (SD) in terms of tumor expression of PD-L1, assessed by immunohistochemistry, before the initiation of anti-PD(L)1 therapy.

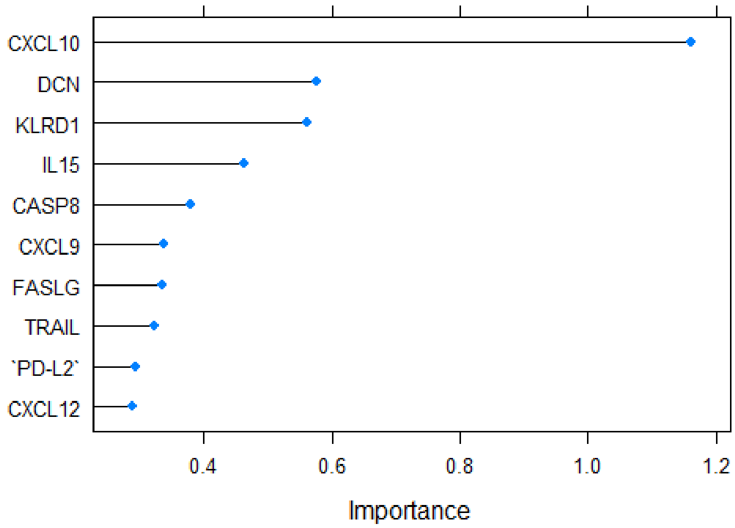

Figure S6. The top 10 proteins with the highest discrimination power in the random forest model.

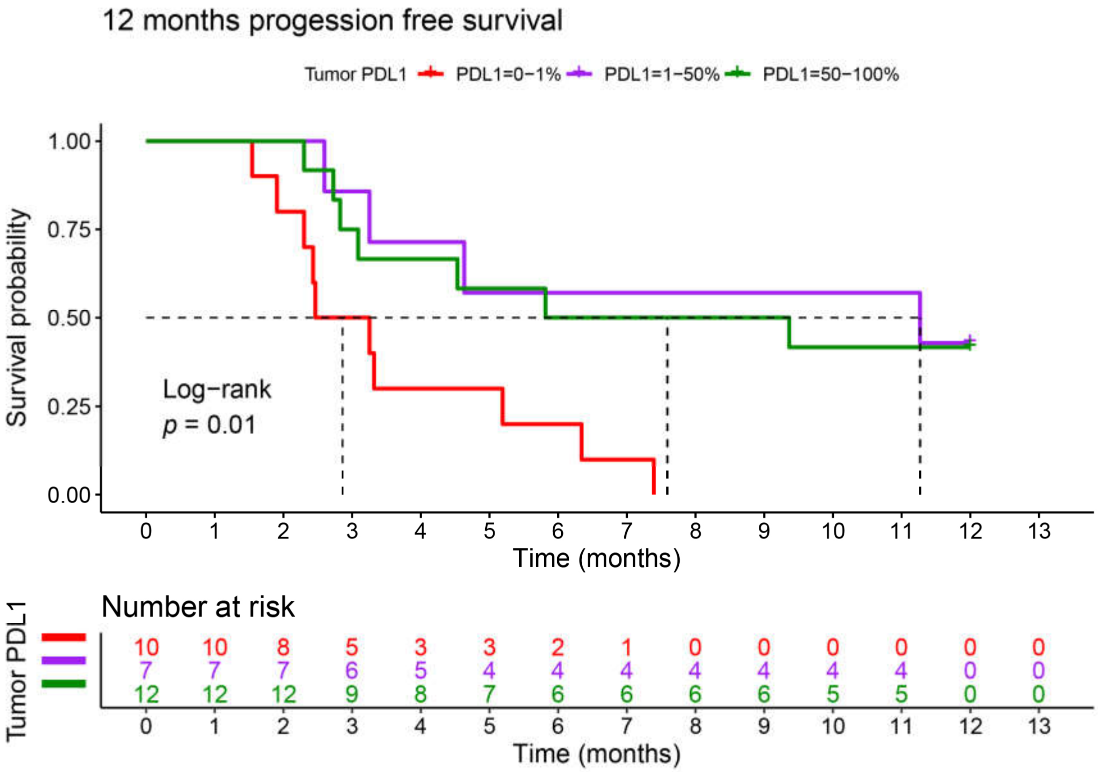

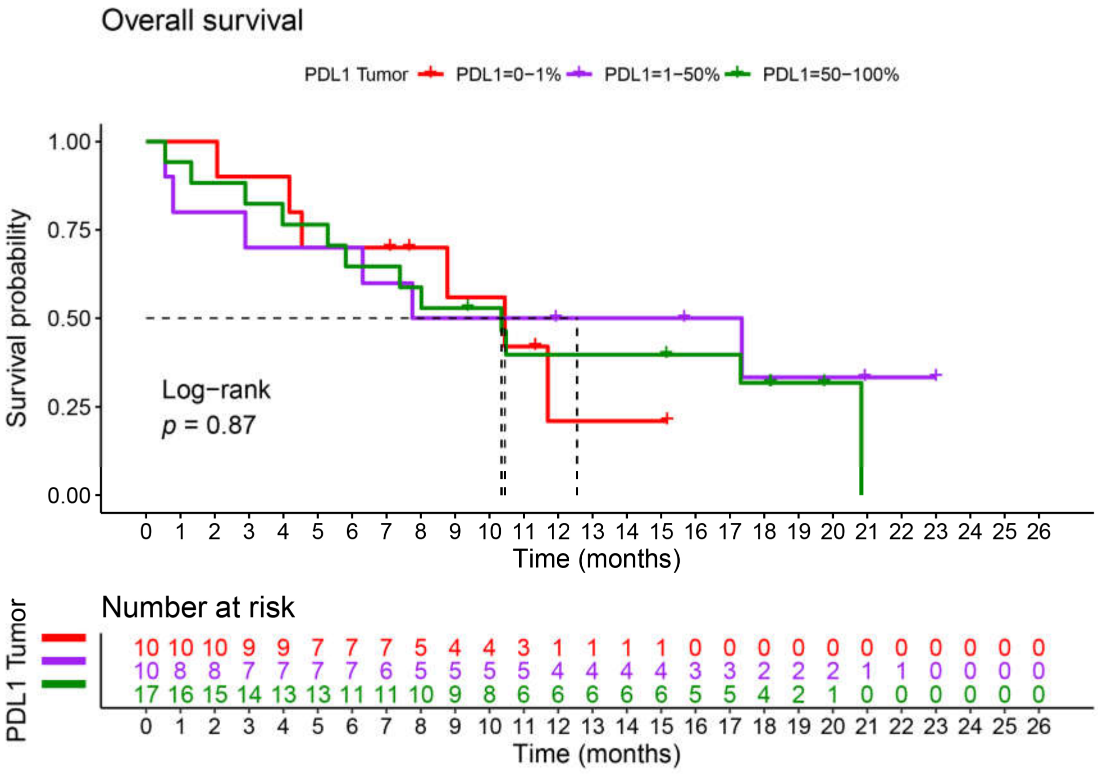

Figure S7. The association between progression-free survival (PFS) and overall survival (OS) and with the tumor PD-L1 expression. Kaplan–Meier curves of PFS and OS according to tumor PD-L1 expression groups (0-1%, 150% and 50-100%) are presented. Differences in survival between the groups were calculated by log rank test.

Table S1. Plasma proteins assessed with the multiplex proteomic analysis. The mean, median and standard deviation of the NPX value for each protein are displayed.

#	Biomarker	Mean	Median	Standard deviation
**1**	ADA	5.207	5.106	0.4876
**2**	ADGRG1	2.623	2.317	1.224
**3**	ANGPT1	9.029	9.198	1.003
**4**	ANGPT2	5.788	5.881	0.4291
**5**	CAIX	5.005	5.081	0.7626
**6**	CASP-8	5.168	5.059	0.7809
**7**	CCL17	9.834	9.803	1.067
**8**	CCL19	9.164	9.208	1.049
**9**	CCL20	7.606	7.498	1.018
**10**	CCL23	10.23	10.32	0.6841
**11**	CCL3	5.933	5.982	0.5311
**12**	CCL4	6.531	6.665	0.4534
**13**	CD244	6.28	6.203	0.3903
**14**	CD27	7.935	7.908	0.5552
**15**	CD4	3.509	3.482	0.3435
**16**	CD40	10.16	10.15	0.4434
**17**	CD40-L	7.08	7.281	1.463
**18**	CD5	5.016	5.027	0.4071
**19**	CD70	3.753	3.827	0.7026
**20**	CD83	2.738	2.774	0.469
**21**	CD8A	9.224	9.245	0.6812
**22**	CRTAM	5.611	5.432	0.7388
**23**	CSF-1	9.605	9.646	0.226
**24**	CX3CL1	6.118	6.059	0.4415
**25**	CXCL1	8.924	9.07	0.9046
**26**	CXCL10	10.31	10.24	0.9831
**27**	CXCL11	9.258	9.154	1.337
**28**	CXCL12	0.7573	0.7392	0.2913
**29**	CXCL13	9.058	8.972	0.8978
**30**	CXCL5	11.11	11.23	1.11
**31**	CXCL9	8.443	8.416	0.9723
**32**	DCN	3.953	3.959	0.3928
**33**	EGF	9.385	9.537	1.284
**34**	FASLG	5.278	5.425	0.668
**35**	FGF2	1.515	1.506	0.8661
**36**	Gal-1	6.717	6.735	0.2355
**37**	Gal-9	7.509	7.475	0.3624
**38**	GZMA	6.959	6.935	0.6409
**39**	GZMB	5.324	5.346	0.8977
**40**	GZMH	5.07	5	0.9599
**41**	HGF	9.177	8.951	0.8073
**42**	HO-1	11.19	11.24	0.4382
**43**	ICOSLG	4.788	4.794	0.2633
**44**	IFN-gamma	5.668	5.453	1.26
**45**	IL10	3.779	3.389	1.297
**46**	IL12	6.755	6.983	1.096
**47**	IL12RB1	1.483	1.426	0.4478
**48**	IL15	4.161	4.102	0.348
**49**	IL18	8.945	8.891	0.7163
**50**	IL6	4.437	4.64	1.452
**51**	IL7	4.557	4.632	0.9291
**52**	IL8	6.457	6.198	0.8853
**53**	KIR3DL1	1.218	1.214	1.316
**54**	KLRD1	6.566	6.559	0.7708
**55**	LAG3	2.213	2.007	0.6784

**56**	LAMP3	4.842	4.976	0.7136
**57**	LAP TGF-beta-1	9.114	9.113	0.702
**58**	MCP-1	10.7	10.76	0.4852
**59**	MCP-2	7.673	7.637	0.6686
**60**	MCP-3	2.341	2.298	0.7292
**61**	MCP-4	9.362	9.38	0.765
**62**	MIC-A/B	4.013	4.594	1.759
**63**	MMP12	8.066	7.982	1.391
**64**	MMP7	9.503	9.532	0.3827
**65**	MUC-16	5.045	4.672	1.77
**66**	NCR1	3.507	3.503	0.473
**67**	NOS3	1.647	1.582	0.6614
**68**	PDCD1	4.12	4.09	0.5776
**69**	PDGF subunit B	9.753	9.954	0.4797
**70**	PD-L1	5.633	5.669	0.5513
**71**	PD-L2	1.99	1.957	0.3543
**72**	PGF	8.266	8.202	0.4961
**73**	PTN	4.295	4.115	1.282
**74**	TIE2	7.042	7.099	0.289
**75**	TNF	2.995	2.902	0.9964
**76**	TNFRSF12A	4.942	4.808	0.5389
**77**	TNFRSF21	7.269	7.218	0.264
**78**	TNFRSF4	5.68	5.636	0.5821
**79**	TNFRSF9	6.122	6.138	0.8479
**80**	TNFSF14	4.79	4.587	0.883
**81**	TRAIL	8.254	8.241	0.3355
**82**	TWEAK	8.616	8.492	0.5121
**83**	VEGFA	8.248	8.344	0.677
**84**	VEGFR-2	8.016	8.027	0.2548

NPX= Normalized protein expression.

Table S2. Functional subgroups of the OLINK immuno-oncology proteins to T cell activation-, APC-, myeloid/angiogenesis- and tumor-related protein markers.

T cell activation	APC	Myeloid/angiogenesis	Tumor-related
**TNFRSF9**	CD40	IL8	EGF
**CD27**	CD70	ADA	ADGRG1
**CD4**	CD83	ANGPT1	ANGPT2
**CD40-L**	ICOSLG	ARG1	DCN
**CD8A**	IL12	CCL17	FGF2
**CX3CL1**	IL12RB1	CCL3	HGF
**CXCL10**	LAMP3	CCL4	MUC-16
**CXCL11**	PD-L1	CSF-1	PDGF subunit B
**CXCL9**	PD-L2	CXCL1	PTN
**FASLG**		CXCL12	
**GZMA**		CXCL5	
**GZMB**		Gal-1	
**GZMH**		Gal-9	
**IFN-gamma**		LAP TGF-beta-1	
**IL15**		MCP-1	
**IL7**		MCP-2	
**KLRD1**		MCP-3	
**TNF**		MCP-4	
**TNFRSF21**		MMP12	
**TNFRSF4**		MMP7	
**TNFSF14**		NOS3	
**TRAIL**		PGF	
		TIE2	

TNFRSF12A
VEGFA
VEGFR-2

APC=antigen-presenting cell.

Table S3. Plasma proteins assessed with the multiplex proteomic analysis. The median NPX values for each protein in the immune "hot" and immune "cold" clusters based on T cell activation-related markers are displayed. p-value determined by t-test with correction for multiple testing (FDR).

Rank	Protein	Hot signature median	Cold signature median	*p* value	q value
**1**	CD4	3.716	3.247	3.87×10^{-7}	1.83×10^{-6}
**2**	CXCL10	10.9	9.548	2.62×10^{-7}	1.83×10^{-6}
**3**	CXCL9	8.997	7.744	2.71×10^{-6}	8.53×10^{-6}
**4**	TNFRSF4	6.006	5.268	4.32×10^{-6}	1.02×10^{-5}
**5**	CD27	8.239	7.552	8.62×10^{-6}	1.63×10^{-5}
**6**	GZMA	7.271	6.542	6.66×10^{-5}	0.000105
**7**	CD8A	9.532	8.835	0.000415	0.00049
**8**	GZMH	5.505	4.521	0.000404	0.00049
**9**	TNFRSF9	6.481	5.667	0.001049	0.001078
**10**	GZMB	5.703	4.846	0.001141	0.001078
**11**	KLRD1	6.884	6.164	0.001508	0.001295
**12**	TNF	3.396	2.488	0.002008	0.001581
**13**	IFNg	6.154	5.053	0.003255	0.002366
**14**	CD40-L	6.68	7.585	0.042516	0.028698
**15**	TNFRSF21	7.342	7.178	0.041493	0.028698
**16**	CX3CL1	6.216	5.993	0.100784	0.063494
**17**	FASLG	5.409	5.112	0.150109	0.088658
**18**	TRAIL	8.31	8.184	0.229153	0.127382
**19**	IL7	4.437	4.71	0.343917	0.180556
**20**	TNFSF14	4.707	4.895	0.49519	0.246292
**21**	CXCL11	9.3	9.205	0.819258	0.387099
**22**	IL15	4.159	4.164	0.962634	0.433185

Note that NPS is a Log2 value.

Table S4. Plasma proteins assessed with the multiplex proteomic analysis. The mean and NPX values for each protein in the clinical benefit (partial response and stable disease) and progressive disease patients are displayed. p-value determined by t-test with correction for multiple testing (FDR). Note that NPS is a Log2 value.

Rank	Protein	Mean "clinical benefit"	Mean "progressive disease"	*p* value	q value
**1**	PDL1 Tumor	57.73	13.17	**0.000262**	**0.023022**
**2**	IL15	4.309	3.999	**0.006163**	0.182929
**3**	CXCL10	10.84	9.873	**0.006236**	0.182929
**4**	CASP-8	4.758	5.4	**0.016016**	0.303636
**5**	ADA	4.958	5.365	**0.018657**	0.303636
**6**	CXCL9	8.828	8.127	**0.049878**	0.521184
**7**	PGF	8.431	8.095	0.051795	0.521184
**8**	MCP-3	2.493	2.044	0.058539	0.521184
**9**	Gal-9	7.617	7.391	0.065608	0.521184
**10**	IFN-gamma	6.166	5.469	0.068418	0.521184
**11**	DCN	4.149	3.845	0.06970	0.303636
**12**	TNFRSF4	5.87	5.503	0.07107	0.521184
**13**	MUC-16	4.308	5.356	0.087766	0.541064
**14**	TNFRSF12A	5.099	4.77	0.091075	0.541064
**15**	CD70	3.984	3.573	0.105971	0.541064
**16**	HO-1	11.11	11.35	0.10808	0.541064
**17**	HGF	9.371	8.941	0.11093	0.541064
**18**	CXCL12	0.6879	0.8437	0.116672	0.541064
**19**	CX3CL1	6.278	6.033	0.116821	0.541064
**20**	PTN	4.761	4.037	0.123117	0.541715
**21**	PD-L2	2.098	1.913	0.150863	0.573343
**22**	ADGRG1	2.887	2.276	0.15439	0.573343
**23**	TNF	3.25	2.711	0.155873	0.573343

**24**	TNFRSF21	7.307	7.189	0.166476	0.573343
**25**	MCP-1	10.86	10.62	0.170468	0.573343
**26**	CXCL13	9.291	8.838	0.172898	0.573343
**27**	CD4	3.581	3.433	0.176223	0.573343
**28**	CCL3	6.032	5.78	0.182427	0.573343
**29**	CD244	6.144	6.311	0.20072	0.602436
**30**	VEGFR-2	7.967	8.088	0.205376	0.602436
**31**	IL12RB1	1.515	1.348	0.239934	0.665457
**32**	LAG3	2.326	2.038	0.241984	0.665457
**33**	ANGPT2	5.868	5.714	0.271646	0.689068
**34**	CD27	8.005	7.79	0.278714	0.689068
**35**	TRAIL	8.356	8.241	0.279761	0.689068
**36**	CD40-L	6.74	7.3	0.281891	0.689068
**37**	NCR1	3.591	3.424	0.310299	0.719422
**38**	NOS3	1.654	1.439	0.318955	0.719422
**39**	LAMP3	4.963	4.71	0.324723	0.719422
**40**	MCP-2	7.513	7.736	0.32701	0.719422
**41**	IL18	8.726	8.956	0.344386	0.724964
**42**	TNFSF14	4.589	4.865	0.346006	0.724964
**43**	CD8A	9.294	9.076	0.367658	0.737002
**44**	IL10	3.853	3.475	0.368501	0.737002
**45**	MMP7	9.61	9.496	0.393688	0.76988
**46**	CSF-1	9.644	9.583	0.408107	0.780727
**47**	IL12	6.817	6.547	0.437593	0.819323
**48**	CCL20	7.691	7.42	0.471473	0.839447
**49**	LAP TGF-beta-1	9.013	9.184	0.473007	0.839447
**50**	CD83	2.751	2.649	0.483856	0.839447
**51**	TNFRSF9	6.242	6.027	0.494951	0.839447
**52**	CD40	10.08	10.19	0.502586	0.839447
**53**	ICOSLG	4.839	4.775	0.505576	0.839447
**54**	GZMB	5.475	5.301	0.559537	0.890777
**55**	FGF2	1.364	1.521	0.607358	0.890777
**56**	PD-L1	5.522	5.626	0.607603	0.890777
**57**	CCL17	9.999	9.822	0.624336	0.890777
**58**	CD5	4.962	5.031	0.62522	0.890777
**59**	IL6	4.092	4.315	0.632362	0.890777
**60**	GZMA	6.869	6.974	0.637715	0.890777
**61**	MCP-4	9.481	9.362	0.655205	0.899878
**62**	CAIX	5.062	4.951	0.664682	0.899878
**63**	IL7	4.422	4.554	0.679067	0.905423
**64**	MIC-A/B	3.912	3.657	0.702912	0.909989
**65**	KIR3DL1	1.461	1.275	0.710565	0.909989
**66**	FASLG	5.316	5.388	0.713514	0.909989
**67**	PDGF subunit B	9.733	9.781	0.775351	0.968643
**68**	EGF	9.329	9.457	0.781519	0.968643
**69**	CCL23	10.21	10.16	0.807238	0.97982
**70**	IL8	6.432	6.356	0.812806	0.97982
**71**	TIE2	7.018	7.039	0.839692	0.982517
**72**	VEGFA	8.173	8.214	0.855355	0.982517
**73**	CCL4	6.488	6.515	0.858651	0.982517
**74**	MMP12	8.089	8.003	0.867502	0.982517
**75**	CXCL11	9.279	9.204	0.879076	0.982517
**76**	ANGPT1	9.042	9.086	0.89431	0.982517
**77**	CXCL5	11.17	11.22	0.903486	0.982517
**78**	CXCL1	8.936	8.899	0.907709	0.982517
**79**	PDCD1	4.106	4.084	0.918678	0.982517
**80**	CCL19	9.224	9.191	0.927029	0.982517
**81**	Gal-1	6.741	6.735	0.94756	0.982517

**82**	KLRD1	6.493	6.476	0.952431	0.982517
**83**	TWEAK	8.678	8.684	0.969791	0.982517
**84**	GZMH	5.003	4.991	0.971352	0.982517
**85**	CRTAM	5.54	5.545	0.987367	0.987367
